# HIV Testing in Non-Traditional Settings – The HINTS Study: A Multi-Centre Observational Study of Feasibility and Acceptability

**DOI:** 10.1371/journal.pone.0039530

**Published:** 2012-06-22

**Authors:** Michael Rayment, Alicia Thornton, Sundhiya Mandalia, Gillian Elam, Mark Atkins, Rachael Jones, Anthony Nardone, Patrick Roberts, Melinda Tenant-Flowers, Jane Anderson, Ann K. Sullivan on behalf of the HINTS Study Group

**Affiliations:** 1 Directorate of HIV/GU Medicine, Chelsea and Westminster Hospital NHS Foundation Trust, London, United Kingdom; 2 Health Protection Agency, Centre for Infections, London, United Kingdom; 3 St Stephen’s AIDS Trust, Chelsea and Westminster Hospital NHS Foundation Trust, London, United Kingdom; 4 Centre for Sexual Health & HIV Research, Research Department of Infection & Population Health, University College London, London, United Kingdom; 5 Department of Clinical Virology, Imperial College London, London, United Kingdom; 6 Directorate of HIV/GU Medicine, Chelsea and Westminster Hospital NHS Foundation Trust, London, United Kingdom; 7 Health Protection Agency, Centre for Infections, London, United Kingdom; 8 Department of Emergency Medicine, Chelsea and Westminster NHS Foundation Trust, London, United Kingdom; 9 Department of Sexual Health and HIV, The Caldecot Centre, King’s College Hospital NHS Foundation Trust, London, United Kingdom; 10 Centre for the Study of Sexual Health and HIV, Homerton University Hospital NHS Foundation Trust, London, United Kingdom; 11 Directorate of HIV/GU Medicine, Chelsea and Westminster Hospital NHS Foundation Trust, London, United Kingdom; Tulane University, United States of America

## Abstract

**Background:**

UK guidelines recommend routine HIV testing in healthcare settings if the local diagnosed HIV prevalence >2/1000 persons. This prospective study assessed the feasibility and acceptability, to patients and staff, of routinely offering HIV tests in four settings: Emergency Department, Acute Care Unit, Dermatology Outpatients and Primary Care. Modelling suggested the estimated prevalence of undiagnosed HIV infection in attendees would exceed 1/1000 persons. The prevalence identified prospectively was not a primary outcome.

**Methods:**

Permanent staff completed questionnaires assessing attitudes towards routine HIV testing in their workplace before testing began. Subsequently, over a three-month period, patients aged 16–65 were offered an HIV test by study staff. Demographics, uptake, results, and departmental activity were collected. Subsets of patients completed questionnaires. Analyses were conducted to identify factors associated with test uptake.

**Findings:**

Questionnaires were received from 144 staff. 96% supported the expansion of HIV testing, but only 54% stated that they would feel comfortable delivering testing themselves, with 72% identifying a need for training. Of 6194 patients offered a test, 4105 (66·8%) accepted (61·8–75·4% across sites). Eight individuals were diagnosed with HIV (0–10/1000 across sites) and all transferred to care. Younger people, and males, were more likely to accept an HIV test. No significant associations were found between uptake and ethnicity, or clinical site. Questionnaires were returned from 1003 patients. The offer of an HIV test was acceptable to 92%. Of respondents, individuals who had never tested for HIV before were more likely to accept a test, but no association was found between test uptake and sexual orientation.

**Conclusions:**

HIV testing in these settings is acceptable, and operationally feasible. The strategy successfully identified, and transferred to care, HIV-positive individuals. However, if HIV testing is to be included as a routine part of patients’ care, additional staff training and infrastructural resources will be required.

## Introduction

In 2009, an estimated 26% of all adults living with HIV infection in the UK were thought to be undiagnosed. [1] Of those newly diagnosed, more than half presented at a late stage, with a CD4 count of less than 350 per µl. [1] Late diagnosis of HIV infection is associated with poorer responses to antiretroviral therapy [2], [3] and a worse prognosis. [4] A substantial number of HIV-infected individuals will present to health care providers with symptoms attributable to HIV infection in the period preceding their diagnosis, without the underlying diagnosis being made. [5] Beyond the value to the individual, efforts to reduce the undiagnosed fraction may have public health benefits. An estimated 50% of new HIV infections in the US are transmitted by the 25% of HIV-positive individuals unaware of their status. [6] Knowledge of HIV status reduces risk behaviours [7] and effective antiretroviral therapy reduces transmission risk: thus earlier diagnosis is likely to reduce HIV transmission at a population level. [8].

Guidance from the National Institute for Health and Clinical Excellence (NICE) joins existing recommendations from the Chief Medical and Nursing Officers, the British Association for Sexual Health and HIV, the British HIV Association and the British Infection Society in calling for the expansion of HIV testing to settings beyond sexual health clinics and antenatal care. [9]–[12] One recommendation is that HIV testing should be offered to all general medical admissions and primary care registrants in areas where the diagnosed prevalence of HIV infection exceeds 2 per 1000 persons. Thirty-seven Primary Care Trusts exceed this prevalence threshold. [1].

Routine HIV testing in antenatal care and sexual health clinics in the UK has greatly increased HIV testing rates. [1], [13] Routine testing has been shown in an RCT to be acceptable to patients and to cause no undue anxiety. [14] In the US, the CDC has recommended routine testing for all individuals aged 13 to 64 presenting to any healthcare facility since 2006. [15] In those States that have implemented this guidance, the number of new HIV diagnoses has risen, and the proportion of individuals diagnosed late has fallen. [16] No data exists in the UK regarding how such programmes may function within the health service. The HIV Testing in Non-traditional Settings (HINTS) study was conceived to investigate the feasibility and acceptability, to patients and staff, of delivering routine HIV testing in non-specialist settings, in areas of high HIV prevalence.

## Methods

### (1) Clinical Sites, Study Participants and Methods

The HINTS study was an observational study conducted at four locations in London, UK: an Emergency Department (ED), an Acute Care (medical and surgical admissions) Unit (ACU), a Dermatology Outpatient Clinic (OPD), and a Primary Care Centre (PC). All are located within areas of diagnosed HIV prevalence >2/1000 persons as per the Health Protection Agency’s *Survey of Prevalent HIV Infections Diagnosed* (SOPHID) dataset (Table 1). Pre-study modelling using this dataset, combined with estimated HIV-undiagnosed fractions from national unlinked anonymous seroprevalence surveys, demonstrated that the estimated prevalence of undiagnosed HIV infection amongst attendees in all four settings would exceed 1/1000 persons - the suggested cost effectiveness threshold in UK guidelines (data not shown).

**Table 1:** HINTS Study Sites.

Study Site	Primary Care Trust (PCT)	Diagnosed HIV prevalence per 1000 individuals 15–59 years living in PCT (ranking in UK) [1]	Model of routine HIV testingservice delivery: *Composition of* *HIV testing team* (TT)*Delivery model* (DM)	Testing phase: weeks (dates conducted)	HIV testing method: *Sample* (S) *Assay* (A) *Platform* (P) *Confirmation* (C)
Chelsea and WestminsterHospital EmergencyDepartment (ED) *(45 000* *attendances/annum)*	Kensington and Chelsea	8·33 (4^th^)	**TT:** seconded staff from ED,Sexual Health, non-clinical testers	15 (August-November 2009)	**S:** Oral fluid (whole saliva sample)
			**DM:** two testers per testing shifton a rolling rota; planned coverageof all time periods and days		**A:** Bio-Rad Genscreen Ultra HIV Ag-Ab EIA test (Bio-Rad laboratories, Hertfordshire, UK)
					**P:** Biokit Best 2000 (Launch Diagnostics, Kent, UK)
					**C:** Abbott Determine HIV 1/2 test (Abbott Diagnostics, Berkshire, UK)
Homerton UniversityHospital - Acute Care Unit(**ACU**) *(4200 attendances/annum)*	City and Hackney	8·25 (5^th^)	**TT:** seconded staff from mixedbackgrounds (ED, Sexual Health,non-clinical testers, research nurse)	12 (January-April 2010)	**S:** Whole serum
			**DM:** single tester reviewed electronicbed state once daily and approachedall age-eligible patients admittedin the preceding 24 hours,still present in the ACU		**A:** Abbott Architect HIV Ag/Ab 4^th^ generation assay (Abbott Diagnostics, Berkshire, UK)
					**P:** Abbott Architect ci8200 Integrated System (Abbott Diagnostics, Berkshire, UK)
					**C:** Confirmation on repeat
Kings College Hospital - Dermatology Outpatient Department (**OPD**) *(5000 attendances/annum)*	Lambeth	13·28 (1^st^)	**TT:** seconded staff from mixed backgrounds (dermatologists, Sexual Health, non-clinical testers, medical students)	12 (July–September 2010)	**S:** Oral fluid (sample collection with Oracol+ device, Malvern Medical Developments PLC, Worcestershire, UK)
			**DM:** two testers present in each Clinic. Patients offered HIV test prior to Dermatology consultation		**A:** Bio-Rad Genscreen Ultra HIV Ag-Ab EIA test (Bio-Rad laboratories, Hertfordshire, UK)
					**P:** TECAN RMP200 (Tecan UK Ltd, Berkshire, UK)
					**C:** Confirmation on repeat
North End Medical Centre - Primary Care (**PC**) *13 671 registered patients aged 16–65 (2010)*	Hammersmith and Fulham	8·15 (6^th^)	**TT:** all GPs and nurse practitioners in the practice who had received focussed training	14 (February-May 2010)	**S:** Oral fluid (sample collection with Oracol+ device, Malvern Medical Developments PLC, Worcestershire, UK)
			**DM:** testing introduced to routine consultations in a staggered fashion		**A:** Bio-Rad Genscreen Ultra HIV Ag-Ab EIA test (Bio-Rad laboratories, Hertfordshire, UK)
					**P:** Biokit Best 2000 (Launch Diagnostics, Kent, UK)
					**C:** Abbott Determine HIV 1/2 test (Abbott Diagnostics, Berkshire, UK)

[1] Health Protection Agency. HIV in the United Kingdom 2010. Health Protection Agency; 2010 [accessed 24th January 2011]; Available from: http://www.hpa.org.uk/Publications/InfectiousDiseases/HIVAndSTIs/1011HIVUK2010Report/.

The study was divided into two phases. During the pre-study phase, a questionnaire was administered to all clinical and support staff within each department, assessing attitudes and knowledge towards HIV infection and the provision of HIV testing in non-specialist settings. All staff were encouraged to respond, and the return rate was calculated. The majority of staff completing the questionnaire were not directly involved in delivering the subsequent HIV testing programmes (see below).

During the study phase, HIV testing was offered to all sequential patients accessing the healthcare setting, who fulfilled the inclusion criteria, as follows: aged between 16 and 65 years and (i) not known to be HIV positive, (ii) accessing the healthcare setting for the first time over the testing period, and (iii) able to consent to a test. A leaflet was provided and verbal consent was obtained prior to HIV testing. Delivery of HIV testing in these settings was in line with published National guidelines, [10] and as such, verbal consent only to an HIV test was deemed sufficient, and in line with good clinical practice in the UK. The leaflet was available in multiple languages. In the ED, ACU and OPD, tests were offered by super-numerary study staff. These staff comprised seconded local sexual health and permanent departmental staff, and a small number of non-clinical staff. In the PC arm, testing was delivered by general practitioners during clinical consultations. All staff offering tests had received focussed didactic and practical training in the provision of HIV tests before the study began (see Table 1). Results governance and delivery was managed by the local Sexual Health service. Patients with a reactive HIV test were recalled to undergo confirmatory HIV testing. A helpline number was provided, and sexual health counsellors were available to all patients upon request.

Oral fluid-based HIV testing was used in the PC, ED and OPD arms, and was performed using a fourth-generation assay on a modified platform to detect HIV-1 antibodies. Internal validation of this technique in the pre-study phase showed it to be robust. [17] In the ACU, fourth generation HIV serology was performed on serum samples obtained during the inpatient admission (Table 1).

Questionnaires were administered to sequential patients offered HIV tests at prospectively planned intervals throughout the testing period, in order to minimise selection bias. The questionnaire distribution strategy was designed in a pragmatic fashion, in order to maximise the amount of time during which HIV testing was the only additional intervention. As provision of the questionnaire required additional time, it impacted upon the number of tests that could be offered; therefore questionnaires were offered only on pre-determined days. On these days, questionnaires were sequentially offered to all patients who were eligible for the study (both those who accepted testing and those who declined). The questionnaire collected basic demographic data, HIV testing behaviour, and attitudes towards a series of statements on HIV testing, quantified by modified Likert scales. In the PC arm, active recruitment to the questionnaire study was also supplemented retrospectively by a mobile phone text recruitment campaign.

### (2) Ethics

Full, verbal consent was sought prior to HIV testing, in line with good clinical practice (as per General Medical Council guidance) and in line with the published, national, clinical guidelines. [10] Written consent to test was thus deemed unnecessary and viewed as a potential disincentive to test. The outcome of the test offer was recorded in the case notes. Questionnaires were fully anonymous and verbal consent was sought prior to completion. A favourable ethical opinion to conduct the research in the manner described above was obtained from the UK National Research Ethics Service (REC reference: 09/H0805/35) which confers freedom to conduct the research across all National Health Service settings in the UK, subject to local Research and Development Office registration. Such registration to conduct the research was obtained in all four National Health Service settings: Chelsea and Westminster NHS Foundation Trust, London; Homerton University Hospital Foundation Trust, London; Kings College Hospital NHS Foundation Trust, London; North End Medical Centre, NHS Hammersmith and Fulham, London (Table 1).

### (3) Primary Outcomes

The primary outcome measures of the study comprised:

- feasibility of delivering routine HIV testing in the non-specialist setting, as evaluated by measuring the proportion of eligible individuals offered tests
- acceptability, to patients and staff, of screening method and site, as evaluated by test uptake and the results of the patient and staff questionnaires
- proportion of individuals newly diagnosed with HIV infection in non-specialist settings successfully transferred to specialist HIV clinical care

### (4) Sample Size and Data Capture

A modelling exercise (described in the Methods section) was undertaken to estimate the prevalence of undiagnosed HIV in the populations using each setting. At a projected 2/1000 persons, measuring the true prevalence of undiagnosed HIV in the study settings to within +/−0·05% would require sampling of >30 000 subjects. Determining true prevalence was therefore not a primary outcome of the study. In order to answer our primary outcomes of feasibility of delivering testing, and of staff and patient acceptability, it was decided to time-limit the trial, with a minimum of twelve weeks testing at each site. We projected reaching 8000 patients for testing, and administering 1000 patient questionnaires.

Electronic patient records were used to capture age, sex, ethnicity, reasons for non-offer of HIV test (including ineligibility) and the outcome of the test offer. All data were anonymised prior to analysis. Data were held in line with the Data Protection Act 1998 and local Trust policies.

### (5) Statistical Analysis

Operational (feasibility) data (populations attending, test offer rate, test uptake) were analysed by site, and differences were assessed for associations using the χ^2^–test, with Yates’ correction applied where appropriate.

Univariate logistic regression analysis was used to examine factors that were associated with the likelihood of test uptake, and this was stratified by clinical site, as differences in accepting an HIV test were found by site. The strata specific adjusted estimates found to be significant (p<0·2) in the univariate model were used to derive a multivariable logistic regression model. In order to ensure no degrees of freedom were lost when building a multivariable model, all variables with missing data were coded with a separate category. The final multivariable model presented has been stratified by clinical site. Stratified logistic regression analyses were repeated, based on data from the subset of patients who completed the questionnaire (the questionnaire collected additional variables: previous HIV testing behaviour and self-reported sexuality).

### (6) Clinical Trial Registration

This study was registered on ClinicalTrails.gov with the Study ID: NCT01138878, in line with local requirements.

## Results

### (1) Staff Attitudes Towards HIV Testing

Pre-study staff questionnaires were completed by 146 members of staff in each of the departments (57 doctors, 56 nurses, 14 allied health professionals and 19 clerical staff). The return rate was: ED 91%; ACU 76%; OPD 82%; PC 92%. Staff members in all departments were supportive of the provision of HIV tests in non-specialist settings and of the provision of HIV testing within their department (Table 2). However, a smaller proportion of doctors and nurses felt comfortable offering the HIV test to patients themselves (ED 57%; ACU 42%; PC 75%; OPD 63%). Analysis of topic areas suggested that the majority of staff at all four sites felt they would require additional training before routinely offering HIV tests to patients (overall: 72% (95%CI 64–80%)). A smaller proportion identified operational barriers, such as a lack of time.

**Table 2:** Staff attitudes and barriers to the provision of HIV tests in non-specialist settings.

	Proportion agreeing with statement (maximum number of respondents to question) [95% CI]				
	ACU (41)	ED (71)	PC (14)	OPD (18)	OVERALL (144)
**Attitudes**					
“HIV testing should be available in services other than sexual health and antenatal clinics”	98%	94%	100%	100%	96% [93–99]
“HIV testing should be routinely offered to everyone”	81%	84%	87%	83%	83% [77–89]
“Offering HIV testing to all patients in this department is a good idea”	79%	80%	79%	83%	80% [73–86]
“I would feel comfortable offering HIV testing to all patients in this department”	42%	57%	75%	63%	54% [45–63]
**Barriers**					
“I don’t think this department provides enough privacy to routinely offer an HIV test to all patients”	44%	58%	0%	17%	43% [35–51]
“I am concerned that patients would have questions I could not answer”	52%	72%	57%	53%	63% [55–70]
“I don’t have time to include routine HIV testing as part of patients’ care in this department”	40%	53%	38%	27%	44% [45–53]
“I would require additional training before routinely offering HIV tests to patients”	65%	82%	56%	63%	72% [64–80]

### (2) HIV Testing

#### (2.1) Populations offered testing

**Figure 1:**
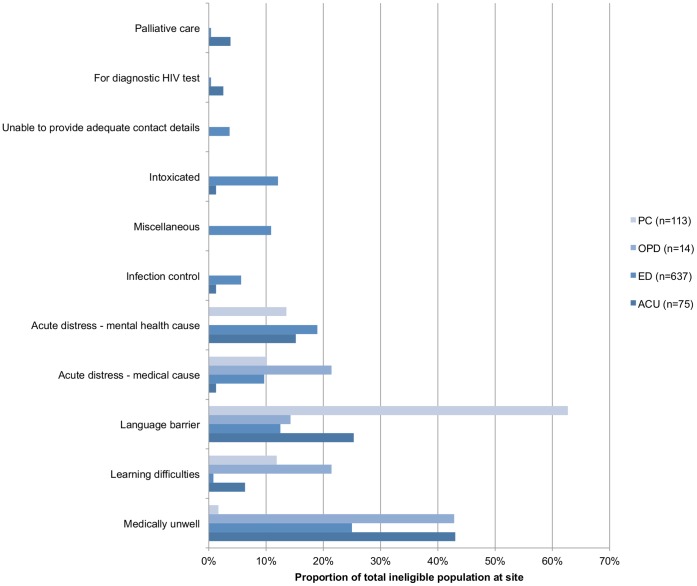
Reasons for clinical ineligibility to participate by site (proportion of total ineligible (%)).

Across all four sites, there were 13 855 age eligible attendees, of whom 7033 (50·7%) were approached by study personnel (Table 3). Of this number, 839 (11·6%) were subsequently considered ineligible to test. The reasons for clinical ineligibility to test across the sites are shown in Figure 1, as proportions of the total at each site. Significant differences were seen in both the approach rate and proportion clinically ineligible to test across the sites (p<0.05 for both comparisons). Differences were also observed between the populations of patients approached for testing, versus the entire populations of eligible patients attending. With respect to the total eligible population, patients approached were more likely to be male (43·9% vs. 37·0%), and less likely to be of Black African origin (5·0% vs. 7·2%) (p<0·05 for both comparisons). Analysis of data by site indicates this was mainly driven by differences observed in the PC and ED arms (data not shown).

**Table 3:** Coverage, uptake and seropositivity of routine offer of HIV test across the four sites.

	ED	ACU	PC	OPD
**Age eligible attendees** ***(n = 15 042)***	5541	1388	6337	1776
**Total study eligible** **attendees (first offer of** **test; not known** **HIV-positive) (%)** ***(n = 13 855)*** ** [95% CI]**	5505 (99·3%) [99·1%–99·6%]	1298 (93·5%) [92·2%–94·8%]	5352 (84·5%) [83·6%–85·4%]	1700 (95·9%) [94·8%–96·7%]
**Coverage = eligible** **attendees approached (%)** ***(n = 7033)*** ** [95% CI]**	4070 (73·9%) [72·7%–75·1%]	623 (48·0%) [45·3%–50·8%]	1442 (26·9%) [25·8%–28·1%]	898 (52·8%) [50·5%–55·2%]
**Clinically ineligible** **(% of all approached)** ***(n = 839)*** ** [95% CI]**	637 (15·7%) [14·5%–16·8%]	75 (12·0%) [9·5%–14·6%]	113 (7·8%) [6·5%–9·2%]	14 (1·5%) [0·8%–2·4%]
**Total tests offered ** ***(n = 6194)***	3433	548	1329	884
**Uptake = total tests** **accepted (%)** ***(n = 4105)*** ** [95% CI]**	2121 (61·8%) [60·2%–63·4%]	384 (70·1%) [66·2%–74·0%]	1002 (75·4%) [73·1%–77·7%]	598 (67·6%) [64·6%–70·7%]
**Reactive HIV tests ** ***(n = 15)***	6	4	5	0
**False reactive HIV tests ** ***(n = 7)***	2	0	5	0
**Newly diagnosed** **individuals ** ***(n = 8)***	4	4	0	0
**HIV seropositivity** **(per 1000) [95% CI]**	1·9 [0·05–3·8]	10·1 [0·3–20·5]		
**Proportion transferred** **to care**	100%	100%	n/a	n/a

#### (2.2) HIV testing data

Of the 6194 patients offered an HIV test, 4105 accepted this offer: test uptake  = 66·8% (Table 3). Eight individuals were newly diagnosed with HIV infection, and all were successfully transferred to care (two partners were subsequently diagnosed with HIV infection through partner notification). HIV seropositivity across the sites was 1.9/1000 persons (95% CI 0·6–3·2/1000). Significant differences in seropositivity are noted between sites (p<0.05). There were seven false reactive screening tests (2 in ED and 5 in PC) as confirmed by further serological testing. These all related to oral fluid HIV screening tests. There were no false reactive screening tests in the ACU arm, where serology was performed.

**Figure 2:**
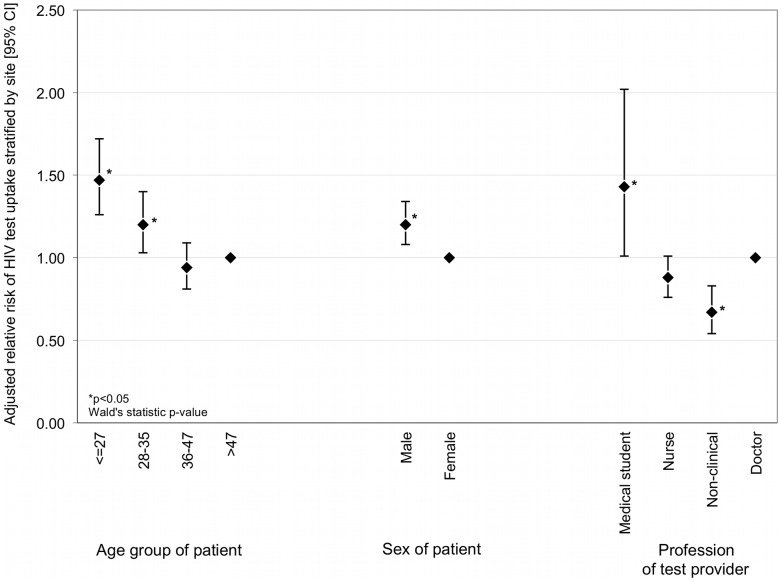
Multivariable logistic regression model showing significant independent predictors of HIV test uptake in patients who were offered the test. *(n = 6194; adjusted for ethnicity, and other variables in the model, and stratified by site).*

Uptake of HIV testing differed significantly by site; range: 61·8%–75·4% (ED vs. PC) (p<0·001). The multivariable model demonstrated that sex, age and the profession of the test provider were significantly associated with test uptake. Male patients and younger patients were more likely to accept tests. Patients were more likely to accept tests when offered the test by a medical student or doctor, rather than by a nurse or a trained tested from a non-clinical background (Figure 2). Site *per se* was not an independent predictor of test uptake.

### (3) Patient Questionnaire Data

#### (3.1) Patient questionnaire respondents

A total of 1003 questionnaires were returned during the testing phase. The number of questionnaires received from each site (n) was not proportional (%) to the total numbers of patients offered testing: ED = 528 (15·3%), ACU = 107 (19·5%), OPD = 297 (33·6%), PC = 71 (5·3%). We are unable to calculate a return rate on questionnaires due to operational differences in the questionnaire study across the sites. In relation to the total population (n = 6194) questionnaire respondents were younger (mean age yrs (SD): 36·0 (12·2) vs. 37·6 (12·8)), more likely to be White (69·9% vs. 56·8%), and more likely to have accepted an HIV test (73·5% vs. 66·8% overall) (p<0·001 for all comparisons).

#### (3.2) Predictors of HIV test acceptance

**Figure 3:**
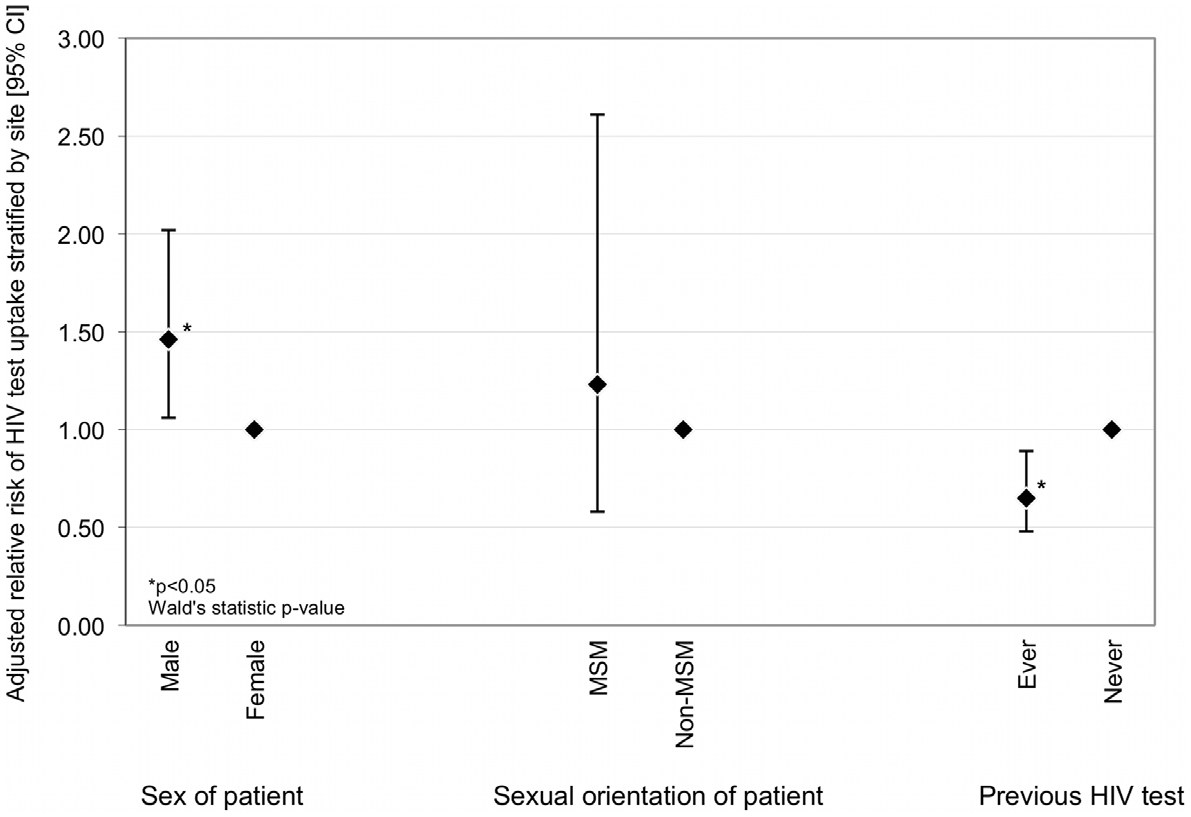
Multivariable logistic regression model showing potential predictors of HIV test uptake in patients who completed the questionnaire. *(n = 1003; adjusted for age, ethnicity and other variables in the model, and stratified by site; MSM = men who have sex with men).*

Using data derived from questionnaire respondents only, previous HIV testing behaviour was shown to be associated with test uptake: 48·5% of respondents had never tested for HIV infection, and this group was more likely to accept an HIV test than those who had previously tested (unadjusted RR 1·00 versus 0·65 (95% CI 0·49–0·87) p = 0·007). A trend was observed amongst previous testers with those having tested recently (less than one year ago) being the least likely to accept an HIV test, and those having tested between two and five years previously the most likely (p<0·001; χ^2^ for trend). The multivariable analysis is shown in Figure 3.

#### (3.3) Reasons for declining an HIV test

Questionnaire respondents who had declined an HIV test (n = 265) were asked to cite reasons for doing so. Respondents could cite more than one reason. The most commonly cited reasons, across all four sites, were: “I have tested recently” (54%), “I do not think I am risk of HIV infection” (47%), and “I have other health concerns today” (24%).

#### (3.4) Patient acceptability of HIV testing

In response to the statement: *“It is acceptable to me to be offered an HIV test in this setting,”* 92% of 1003 questionnaire respondents agreed. Of those accepting an HIV test, this proportion was 97%; in those declining it was 85% (p<0·001). In univariate analysis, greater acceptability was associated with younger age and male sex, plus test uptake. No difference was seen by ethnicity or by clinical site. In multivariable analysis, stratified by clinical site, the acceptability of testing remained positively associated with male sex (RR 2·05 (95%CI 1·12–3·76) p = 0·02) and with younger age groups (<27 yrs RR 2·35 (95%CI 1·03–5·37) p = 0·043). HIV test acceptance was a strong predictor of acceptability (RR in test decliners: 0·22 (95%CI 0·13–0·38) p<0·001). Levels of support to a series of statements advocating the expansion of HIV testing to non-specialist settings were high in both test accepters and test decliners, and were in excess of 90% in all cases.

## Discussion

### What is Already known on this Topic?

The delivery of routine HIV testing in non-specialist medical settings in areas of high HIV prevalence (more than 2 diagnosed cases/1000 persons) is advocated by National Guidelines in the UK. Whilst data exists in the US, there is, to date, no published evidence on the acceptability or feasibility of delivering wide-scale, routine HIV testing in general medical settings within the context of the UK health service. The HINTS Study was conceived to generate an evidence base to inform the development of testing services in the UK.

### What does Our Study Add?

Our study shows that it is acceptable to the majority of patients to be offered an HIV test in a non-specialist setting. Uptake of testing is high across all socio-demographic categories, assuaging concerns that higher risk individuals may be less likely to accept a test in such settings. Patients declining to test still found it acceptable to be offered the test. Many patients accepting a test had never tested for HIV infection before. Cases of undiagnosed HIV were identified, and all patients were successfully transferred to clinical care. Staff members from the departments involved in the study were supportive of the initiative, but many identified educational needs and operational barriers that would need to be addressed if they were to deliver routine HIV testing in the long term.

The HINTS study is the first published data regarding routine, non-targeted testing within secondary care in the UK, and provides evidence on how such a strategy may operate within the UK health service. More than 6000 patients were offered an HIV test, and more than 4100 consented. The uptake of HIV testing across the sites of 66·8% compares favourably with (and in many cases is substantially superior to) uptake rates observed in US settings, and from preliminary reports from other pilot studies in operation in the UK and France. [18]–[22].

The coverage rates are substantially higher than those reported in most US settings. In ED-based programmes in the US, programmes employing near-patient testing augmented by counsellors have managed to reach much smaller fractions (typically <5%) of all eligible patients passing through. [19], [21], [23] The testing intervention in the HINTS Study was brief and augmented by written information. Testing was delivered in line with national guidelines, and lengthy pre-test counselling was not felt to be appropriate within the clinical settings used. Pre-test counselling has not been shown to reduce subsequent HIV transmission risk in a large meta-analysis, [24] but test providers during HINTS were at liberty to provide more intensive counselling on a case-by-case basis. Coverage was likely also increased by not employing near-patient tests, which require clinical expertise and time, and may not sit well within the time-pressured environments of the NHS. Near-patient tests have been associated in US settings with better transfer to care rates and greater acceptability to patients. [21], [25] Our transfer to care rate was 100%. This was likely a function of the co-operation between the testing site and the local Sexual Health service. In the OPD and ACU sites, the population of patients offered HIV tests was representative of the entire population. This suggests no targeting was evident at these sites. In the PC and ED arms, targeting of male patients appears evident, and fewer than expected tests were offered to patients of Black African origin. This is an interesting observation with respect to the ethnic disparity, as it contradicts the expectation that if targeting were present, it would be directed towards those who belong to higher risk populations.

There are several differences between the sites that merit special mention. The seropositivity of previously undiagnosed HIV is significantly different across the sites, with 1.9/1000 and 10.1/1000 HIV-infected persons identified in the ED and ACU settings, and no new diagnoses made in the OPD and PC settings. It is essential to interpret these figures with due caution, as seropositivity was not a primary outcome of the study (as it was not possible to sufficiently power the study to demonstrate this with statistical confidence) but the significant difference between the ED and ACU populations is of interest; it may be related to the acute nature of the settings and the possibly greater severity of presenting complaints, and certainly warrants further investigation. Differences in prevalence across healthcare settings may have clinical and cost benefit implications. The higher case finding rate in inpatient settings may be tempered by differences in the clinical status of patients diagnosed here, as evidenced by UK surveillance data which demonstrate that patients diagnosed as inpatients have lower median CD4 counts and worse outcomes. [1].

The proportions of patients offered testing and the proportions deemed ineligible to participate are also different. This may reflect differences in the patient populations attending each setting, but may also reflect operational differences such as different staffing arrangements. Large patient throughput in the ED, for example, meant two testers needed to be present to offer testing at all times, whereas in the ACU a lone tester making a daily round was deemed sufficient (see Table 1 for details). The coverage in the ACU was limited, however, by the short median stay on the unit: this meant many patients had been discharged before the HIV test offer could be made. Greater proportions of patients were deemed ineligible to test in the acute settings of the ED and ACU, with respect to the non-acute OPD and PC settings: greater numbers of patients were too medically unwell to consent to a test, for example. In the ED, insurmountable language barriers precluded testing in a number of patients, reflecting the particular characteristics of this patient population, and despite our offering of pre-test literature in a number of languages and the availability of interpretation services.

Finally, there were seven false reactive HIV screening tests, all related to the use of oral fluid. With a total of 11 reactive oral fluid tests of 3717 oral fluid tests done, the specificity of the oral fluid test is 99.8% with a positive predictive value of 36%. The positive predictive value will remain a function of the prevalence of true HIV infection in the population tested, and both this figure and the test specificity are acceptable in this context. All patients with reactive screening tests were successfully informed of their results, and all but one returned had their confirmatory testing undertaken in local sexual health service. All of this group found this process acceptable.

Concerning HIV test uptake, the two adjusted models (overall data and questionnaire respondents only) show age, sex, previous testing behaviour and test provider to be independent predictors of likelihood of test uptake, when stratified by clinical site. However, uptake across all socio-demographic categories was good, specifically amongst MSM and minority ethnic groups. This assuages our concerns that higher risk individuals may be less likely to accept an HIV test in a non-specialist setting. Never having previously tested was also positively associated with test uptake. This suggests offering HIV tests in non-specialist settings brings the opportunity of an HIV test to a population who might not otherwise present for one, and that this group welcomes the opportunity. It is important not to assume that patients who decline an HIV test do so because they find it unacceptable. Many patients declining an HIV test did so for understandable reasons. There remained a significant proportion of test decliners who felt that they “were not at risk of HIV infection” and this merits investigation in future work. However, the majority of questionnaire respondents (92%) found the offer of an HIV test in the non-specialist setting acceptable, including 85% of test decliners. Few differences in attitude were observed between test accepters and decliners. The overall level of acceptability is greater than the 72% reported by Drayton *et al*’s survey of patient attitudes to routine testing in the UK. [26].

Whilst staff are accepting of expanding HIV test provision, barriers to implementation at the staff level are evident. Some of the barriers identified are operational in nature, and echo those identified in US ED-based research. [27], [28] Concerns include a lack of time to conduct testing and being ill-prepared to answer patient queries. Testing in this study was delivered in all but one setting (PC) by seconded staff. We cannot therefore generalise many of our operational findings to a service delivery model where testing is offered as part of routine clinical duties. The close working with Sexual Health services, particularly in relation to results governance, recall and transfer to care, was effective, robust and greatly valued by local staff.

There are limitations to the study. Targeting of test offer at the PC and ED sites may have introduced selection bias, which the multivariable analyses and per-site stratification cannot eliminate. We are unable to report a return rate on the questionnaire study, and the differences in the respondent population strongly suggest that questionnaire distribution (and completion) was not systematic. The proportion of questionnaire respondents across the sites, as a fraction of the total number offered testing, was widely different, for example. The questionnaire was available in English only, which may explain some of the ethnic disparity, but other differences are likely to represent offer bias by study staff. As such, generalisability of the questionnaire data is limited. The unadjusted data and adjusted data must be interpreted within the populations studied. The fact that clinical site does not appear to predict test uptake or acceptability of testing in the adjusted analyses suggests that these results could be generalisable in a broader population setting. However, as previously discussed, many of the differences observed between the sites may have been driven by local operational factors, specific and unique to each situation. Broader conclusions and interpretations ought to be limited to the general, cross-site observations that, we believe, confidently demonstrate that testing is acceptable and efficacious. Whilst we believe we have shown that routine HIV testing in non-specialist settings is effective at diagnosing infected individuals, we have yet to prove such an approach is cost effective. This work is ongoing. However, the 2/1000 persons diagnosed prevalence threshold for routine testing advocated in UK guidelines is drawn from US modelling estimates that suggest a lower threshold of 1/1000 persons is likely to be highly cost effective. [29].

In conclusion, the HINTS study demonstrates that routine HIV testing in non-specialist settings in the UK is highly acceptable to patients and staff. Furthermore, it shows such a strategy is feasible to deliver in a diverse range of settings, and is effective at diagnosing HIV-infected individuals and transferring them to care. As such, the study offers a substantial evidence base to underpin strong recommendations from specialist societies, clinical effectiveness bodies, and public health agencies in the UK that routine HIV testing in secondary and primary care settings in areas of high HIV prevalence, ought to be developed and implemented as a matter of urgency. The specific logistics of local implementation will need to be developed within the local context, after appropriate stakeholder engagement.

## References

[1] Health Protection Agency (2010). HIV in the United Kingdom 2010. Health Protection Agency.. http://www.hpa.org.uk/Publications/InfectiousDiseases/HIVAndSTIs/1011HIVUK2010Report/.

[2] Stohr W, Dunn D, Porter K, Hill T, Gazzard B (2007). CD4 cell count and initiation of antiretroviral therapy: trends in seven UK centres, 1997–2003.. HIV Med.

[3] Krentz HB, Auld MC, Gill MJ (2004). The high cost of medical care for patients who present late (CD4<200 cells/microL) with HIV infection.. HIV Med.

[4] Lucas SB, Curtis H, Johnson MA (2008). National review of deaths among HIV-infected adults.. Clin Med.

[5] Sullivan AK, Curtis H, Sabin CA, Johnson MA (2005). Newly diagnosed HIV infections: review in UK and Ireland.. BMJ.

[6] Marks G, Crepaz N, Janssen RS (2006). Estimating sexual transmission of HIV from persons aware and unaware that they are infected with the virus in the USA.. AIDS.

[7] Marks G, Crepaz N, Senterfitt JW, Janssen RS (2005). Meta-analysis of high-risk sexual behavior in persons aware and unaware they are infected with HIV in the United States: implications for HIV prevention programs.. J Acquir Immune Defic Syndr.

[8] Granich RM, Gilks CF, Dye C, De Cock KM, Williams BG (2009). Universal voluntary HIV testing with immediate antiretroviral therapy as a strategy for elimination of HIV transmission: a mathematical model.. Lancet.

[9] Chief Medical Officer letter; Improving the detection and diagnosis of HIV in non-HIV specialties, including primary care.. 2007 [Accessed 24th January 2011].

[10] British Association for Sexual Health and HIV (BASHH), the British HIV Association (BHIVA) and the British Infection Society (BIS) Guidelines for HIV Testing. British HIV Association (2008). Accessed 24 Jan 2011.. http://www.bhiva.org/HIVTesting2008.aspx.

[11] National Institute for Health and Clinical Excellence (2011). Increasing the uptake of HIV testing among men who have sex with men (Guidance: PH34).. London: National Institute for Health and Clinical Excellence.

[12] National Institute for Health and Clinical Excellence (2011). Increasing the uptake of HIV testing among Black Africans in England (Guidance: PH33).. London: National Institute for Health and Clinical Excellence.

[13] Price H, Birchall J, Newey C, Smith F, Waters AM (2009). HIV opt-out increases HIV testing in low-risk patients.. Int J STD AIDS.

[14] Simpson WM, Johnstone FD, Boyd FM, Goldberg DJ, Hart GJ (1998). Uptake and acceptability of antenatal HIV testing: randomised controlled trial of different methods of offering the test.. BMJ.

[15] Branson BM, Handsfield HH, Lampe MA, Janssen RS, Taylor AW (2006). Revised recommendations for HIV testing of adults, adolescents, and pregnant women in health-care settings.. MMWR Recomm Rep 55: 1–17; quiz CE1–4.

[16] Castel A, Samala R, Griffin A, West-Ojo T, Greenberg A (2010). Monitoring the Impact of Expanded HIV Testing in District of Columbia Using Population-based HIV/AIDS Surveillance Data. Conference on Retroviruses and Opportunistic Infections; San Francisco, US.. Paper 34.

[17] Rayment M, Doku E, Thornton A, Nardone A, Sudhanva M (2011). Automated laboratory-based oral fluid testing in HIV-screening programs - “Automatic for the People”? HIV Med..

[18] Brown J, Shesser R, Simon G, Bahn M, Czarnogorski, et al (2007). Routine HIV screening in the emergency department using the new US Centers for Disease Control and Prevention Guidelines: results from a high-prevalence area.. J Acquir Immune Defic Syndr.

[19] Calderon Y, Leider J, Hailpern S, Chin R, Ghosh R (2009). High-volume rapid HIV testing in an urban emergency department.. AIDS Patient Care STDS.

[20] Health Protection Agency. Time to test for HIV: Expanded healthcare and community HIV testing in England. Health Protection Agency (2010). Accessed: 26 Feb 2011.. http://www.hpa.org.uk/Publications/InfectiousDiseases/HIVAndSTIs/1011TimetotestHIVtesting/.

[21] Siegel M, Kennedy L, Rexroth K, Lankford M, Turner M (2011). Better but not ideal acceptance of routine inpatient HIV point-of-care testing among veterans in a high prevalence area.. J Acquir Immune Defic Syndr.

[22] Cremieux A, d’Almeida K, Kierzek G, de Truchis P, Le Vu S (2010). Feasibility and patients’ acceptance of routine HIV screening in 27 Emergency Departments of the Paris urban area, France, May 2009 to August 2010 (ANRS 95008 and Sidaction). Bulletin epidemiologique hebdomadaire. (45–46): 460–3.. *Article in French*.

[23] Jain CL, Jue JS, MacKay R, Wallach F, Factor SH (2008). Acceptance of rapid HIV testing among medical inpatients in New York City.. AIDS Patient Care STDS.

[24] Weinhardt LS, Carey MP, Johnson BT, Bickham NL (1999). Effects of HIV counseling and testing on sexual risk behavior: a meta-analytic review of published research, 1985–1997.. Am J Public Health.

[25] Leider J, Fettig J, Calderon Y (2011). Engaging HIV-positive individuals in specialized care from an urban emergency department. AIDS Patient Care STDS.. Feb.

[26] Drayton R, Keane F, Prentice E (2010). Patients’ attitudes towards increasing the offer of HIV testing in primary and secondary care.. Int J STD AIDS.

[27] Burke RC, Sepkowitz KA, Bernstein KT, Karpati AM, Myers JE (2007). Why don’t physicians test for HIV? A review of the US literature.. AIDS.

[28] Arbelaez C, Wright EA, Losina E, Millen JC, Kimmel S (2012). Emergency Provider Attitudes and Barriers to Universal HIV Testing in the Emergency Department.. J Emerg Med.

[29] Paltiel AD, Walensky RP, Schackman BR, Seage GR 3rd, Mercincavage LM, et al (2006). Expanded HIV screening in the United States: effect on clinical outcomes, HIV transmission, and costs.. Ann Intern Med.

